# Endophthalmitis Requiring Enucleation in a Sedated Critically Ill Patient: A Case Report

**DOI:** 10.7759/cureus.111473

**Published:** 2026-06-25

**Authors:** Malikah Saba

**Affiliations:** 1 Medicine, Queen Elizabeth Hospital Birmingham Charity, Birmingham, GBR

**Keywords:** case report, endophthalmitis, enucleation, infection, trauma

## Abstract

Endophthalmitis is a rare but sight-threatening ophthalmic emergency that may occur following trauma, surgery, or hematogenous spread of infection. Diagnosis may be particularly challenging in critically ill or sedated patients unable to report visual symptoms.

A Caucasian male in his 50s was admitted to the ICU following a motor vehicle collision. During admission, progressive erythematous changes developed around the right eye in the absence of clinically identified ocular trauma, systemic infectious features, or subjective visual symptoms due to ongoing sedation. Computed tomography demonstrated orbital cellulitis with probable endophthalmitis and lens dislocation. Despite urgent ophthalmological intervention, systemic and intravitreal antimicrobial therapy, and orbital decompression, disease progression resulted in corneal perforation requiring enucleation. Early lens dislocation was considered a poor prognostic feature.

This case highlights the diagnostic challenge of rapidly progressive endophthalmitis in sedated critically ill patients and emphasizes the importance of vigilant ocular assessment, early specialist involvement, and prompt treatment to minimize irreversible complications.

## Introduction

Endophthalmitis is an uncommon but vision-threatening ocular emergency that can arise after trauma, surgical procedures, or hematogenous dissemination of infection. Its diagnosis can be especially difficult in critically ill or sedated patients who are unable to communicate visual complaints.

Endophthalmitis is an inflammatory condition involving both the anterior and posterior chambers of the eye [1] and may be classified as infectious or non-infectious. Infectious endophthalmitis is a bacterial or fungal infection involving the vitreous and/or aqueous humor [2], occurring following contamination of the vitreous cavity [3]. Infectious cases may be further classified as exogenous (postoperative or post-traumatic) or endogenous, with most cases being exogenous and occurring following intraocular surgery or ocular trauma. Post-traumatic infectious endophthalmitis accounts for approximately 25-31% of infectious cases [4]. Endogenous endophthalmitis refers to hematogenous metastatic infection arising from a primary infective focus secondary to bacterial or fungal pathology [5].

Common organisms implicated in post-traumatic endophthalmitis include Bacillus species, streptococci, and Gram-negative bacilli, whereas *Staphylococcus aureus* and streptococci are important causes of endogenous endophthalmitis associated with infective endocarditis [2].

Common presenting symptoms of endogenous endophthalmitis include ocular pain, swelling, redness, photophobia, visual disturbance, floaters, and flashes. However, diagnosis may be significantly delayed in critically ill or sedated patients who are unable to report subjective symptoms.

This case highlights the diagnostic challenge posed by rapidly progressive endophthalmitis in sedated critically ill patients, in whom the absence of subjective visual symptoms may delay recognition and treatment despite appropriate nursing eye care. This article was previously posted to the Research Square preprint server on March 5, 2024 [6].

## Case presentation

A Caucasian male in his 50s with no relevant past medical history was admitted to the ICU following a motor vehicle collision. He sustained multiple injuries, including various fractures, bilateral hemopneumothoraces, and liver and splenic lacerations, which were initially managed conservatively.

During his ICU stay, he was intubated due to rapidly worsening respiratory failure and treated for ventilator-associated pneumonia (VAP). He subsequently developed an intrasplenic bleed requiring embolization and a large left-sided pleural collection with associated lung collapse, for which chest drain insertion was required.

Multiple attempts to liberate the patient from mechanical ventilation were unsuccessful, and on day 16, he underwent surgical tracheostomy to facilitate ventilatory weaning.

During this period, erythematous changes and swelling developed around the patient’s right eye. There had been no direct trauma to the eye, no systemic signs of infection, and no subjective visual symptoms could be obtained due to ongoing sedation. Appropriate eye care had been carried out by nursing staff in accordance with institutional practice for sedated critically ill patients, and the affected area was initially monitored closely. However, the rapid progression of the peri-orbital changes prompted urgent ophthalmology review and CT imaging of the orbits.

**Figure 1:**
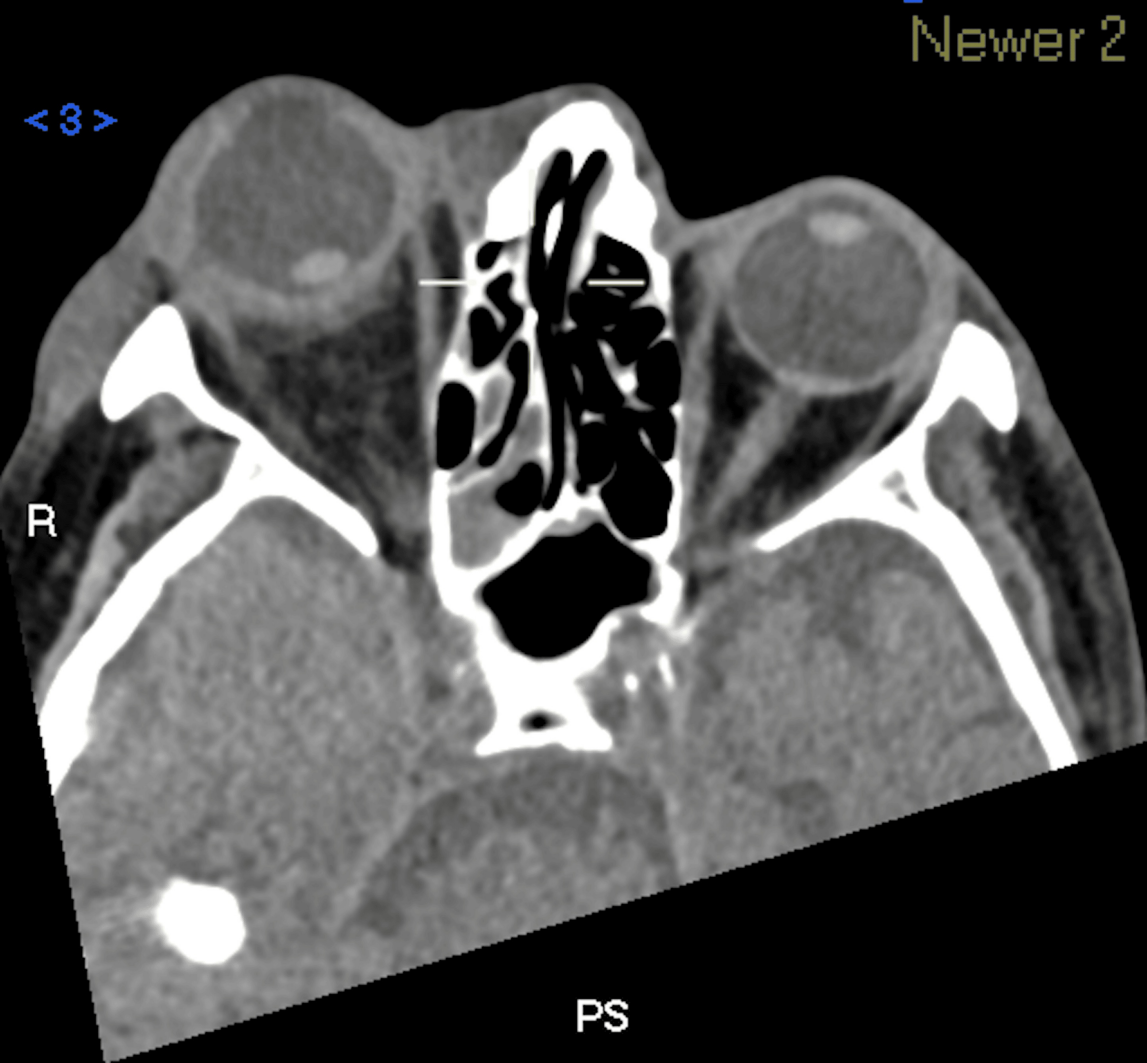
CT imaging demonstrating proptosis, pre-septal swelling, intraconal edema, and possible lens dislocation.

CT imaging demonstrated right orbital cellulitis with probable endophthalmitis and lens dislocation (Figure 1). The patient’s sedation meant that diagnosis relied solely on objective clinical findings, presenting a significant diagnostic challenge.

The patient underwent urgent lateral canthotomy and cantholysis due to rapid disease progression and concern regarding optic nerve compromise, and was commenced on broad-spectrum antimicrobial therapy. This was followed by endoscopic sinus surgery and decompression of the right orbit. Intraoperatively, the orbit was noted to be extremely tense, and complete ocular surface closure proved difficult despite attempted lid apposition for tarsorrhaphy.

Early lens dislocation was considered a poor prognostic feature, and despite surgical intervention together with systemic and intravitreal antimicrobial therapy, the patient developed corneal perforation requiring subsequent enucleation of the right eye. The severity and rapidly progressive nature of the pan-orbital infection, together with evidence of antimicrobial resistance on blood cultures, likely contributed to the poor clinical outcome despite aggressive multidisciplinary management.

## Discussion

Endophthalmitis is an uncommon but sight-threatening ophthalmic emergency that may result in severe visual impairment or loss of the eye if diagnosis and treatment are delayed [2]. This case highlights the diagnostic difficulties encountered in sedated ICU patients, where the absence of subjective visual symptoms may delay recognition of serious ophthalmic pathology.

In critically ill patients, ocular symptoms such as pain, visual disturbance, photophobia, or floaters are often unobtainable due to sedation or reduced consciousness. Consequently, clinicians must rely on objective clinical findings, including periorbital erythema, swelling, intraocular inflammation, or the presence of hypopyon, which may indicate severe ocular infection [7,8]. In this case, rapidly progressive periorbital changes prompted urgent specialist assessment and imaging, leading to diagnosis.

The presence of early lens dislocation was considered an adverse prognostic feature [9] and may reflect severe intraocular disruption associated with advanced disease. Despite prompt ophthalmology involvement, surgical intervention, orbital decompression, and systemic as well as intravitreal antimicrobial therapy, disease progression resulted in corneal perforation requiring enucleation.

Given the rarity and heterogeneity of endophthalmitis, management is frequently guided by clinical presentation, suspected microbiological etiology, and disease severity, with early ophthalmology involvement remaining essential to optimize outcomes.

The Royal College of Ophthalmologists recommends several measures to support eye care in ICU patients, including ocular protection, early identification of ocular pathology requiring urgent specialist review, and appropriate delivery of prescribed ocular therapies [8].

Eye protective measures are based on the degree of ocular exposure [8]. Grade 0 exposure (no exposure): no intervention required; Grade 1 exposure: ocular lubrication; and Grade 2 exposure: lubrication together with eyelid taping or alternative methods of lid closure, including hydrogel or silicone dressings.

This case emphasizes the importance of maintaining a high index of suspicion for ophthalmic complications in sedated ICU patients and highlights the need for vigilant eye assessment, particularly where subjective symptoms cannot be elicited.

## Conclusions

Endophthalmitis is a serious ophthalmic emergency associated with trauma, surgical procedures, and septicemia, requiring rapid diagnosis and treatment to preserve vision. Diagnosis may be particularly challenging in critically ill or sedated patients, where the absence of subjective symptoms can delay recognition.

This case demonstrates how rapidly progressive endophthalmitis may result in devastating consequences despite aggressive medical and surgical management. Early recognition, prompt ophthalmology involvement, appropriate antimicrobial therapy, and meticulous supportive eye care remain essential to minimize irreversible complications, including loss of vision and enucleation.
